# Straight-line orientation in the woodland-living beetle *Sisyphus fasciculatus*

**DOI:** 10.1007/s00359-019-01331-7

**Published:** 2019-04-06

**Authors:** Lana Khaldy, Claudia Tocco, Marcus Byrne, Emily Baird, Marie Dacke

**Affiliations:** 1 Department of Biology, Lund Vision Group, Lund University, Sölvegatan 35, 223 62 Lund, Sweden; 2 School of Animal, Plant and Environmental Science, University of the Witwatersrand, Johannesburg, South Africa; 3 Department of Zoology, Functional Morphology, Stockholm University, Stockholm, Sweden

**Keywords:** Compass, Beetle, Orientation, Sun, Polarised light

## Abstract

To transport their balls of dung along a constant bearing, diurnal savannah-living dung beetles rely primarily on the sun for compass information. However, in more cluttered environments, such as woodlands, this solitary compass cue is frequently hidden from view by surrounding vegetation. In these types of habitats, insects can, instead, rely on surrounding landmarks, the canopy pattern, or wide-field celestial cues, such as polarised skylight, for directional information. Here, we investigate the compass orientation strategy behind straight-line orientation in the diurnal woodland-living beetle *Sisyphus fasciculatus*. We found that, when manipulating the direction of polarised skylight, *Si. fasciculatus* responded to this change with a similar change in bearing. However, when the apparent position of the sun was moved, the woodland-living beetle did not change its direction of travel. In contrast, the savannah-living beetle *Scarabaeus lamarcki* responded to the manipulation of the solar position with a corresponding change in bearing. These results suggest that the dominant compass cue used for straight-line orientation in dung beetles may be determined by the celestial cue that is most prominent in their preferred habitat.

## Introduction

In most environments, the visual surroundings provide an abundance of compass cues that can be used for navigation, migration and orientation. As a distinct feature on clear days, the sun is frequently employed for this purpose (Wehner 1984; Byrne et al. 2003; Guilford and Taylor 2014; Chernetsov 2017). Similarly, the polarised skylight pattern, with the sun at its centre, also provides a reliable reference for guidance (Wehner and Muller 2006; Weir and Dickinson 2012). Light intensity and chromatic gradients, caused by intensity- and wavelength-dependent scattering of sunlight, also serve as compass cues (Rossel and Wehner 1984; Ugolini et al. 2008; el Jundi et al. 2014, 2015a). At night, the moon (Ugolini and Melis 1999), the pattern of polarised moonlight (Dacke et al. 2003a, b, 2011), and the stars (Wiltschko et al. 1987; Mouritsen and Larsen 2001; Mauck et al. 2008; Dacke et al. 2013a; Foster et al. 2017, 2018) are used for the same purpose. In addition, many animals rely on landmarks (Collett 1996), entire skylines (Graham and Cheng 2009; Towne et al. 2017), the earth’s magnetic field (Wiltschko and Wiltschko 1972; Wang et al. 2007; Dommer et al. 2008; Dreyer et al. 2018), odour (Walraff and Foa 1981; Gagliardo et al. 2013), and wind (Mueller and Wehner 2007) to find their way.

The directional information extracted from any set of compass cues can, in principle, be used in three different ways. First, an animal can use a *combination* of terrestrial and celestial cues to find its way to a goal. This can be observed in the Australian desert ants, where the ants head in an intermediate direction when terrestrial and celestial information are set in conflict (Narendra 2007; Collett 2012; Legge et al. 2014). Second, when moving over greater distances and/or over longer periods of time, different compass cues can be used in *sequence* over a length of time. A good example for this can be found in migrating birds, which follow a star compass at night, but use the sun as a compass cue when it appears in the morning sky (Muheim et al. 2003). In the same manner, on clear days, honeybees rely on celestial cues for directional information, but when the sky becomes overcast, these insects will rely primarily on terrestrial cues (Chittka and Geiger 1995). A third approach to compass cue use involves a *‘backup system’* or cue hierarchy (el Jundi et al. 2015b), where the dominant cue will be substituted by a secondary cue when no longer accessible. Pigeons, for example, use the sun as their dominant cue but rely on magnetic cues on overcast days (Walcott 2005). Similarly, most ants will primarily rely on polarised light when navigating but when experimentally deprived of this cue, they will instead orient to their secondary cue, the sun (Wehner and Muller 2006).

All diurnal, savannah-living dung beetles studied to date rely on the sun as their primary cue for orientation when transporting their dung balls away from competitors at the dung pat (Byrne et al. 2003; Dacke et al. 2013b, 2014; el Jundi et al. 2014, 2015b). As soon as the sun is out of view, which may occur due to cloud cover or vegetation, these beetles will instantaneously, with no hesitation in their stride or decrease in orientation performance, change to the next cue in the hierarchy (Byrne et al. 2003). This cue is most likely the celestial pattern of polarised light (Byrne et al. 2003; el Jundi et al. 2014). If met with a condition where neither the sun nor the polarised skylight is available, diurnal dung beetles rely on the skylight intensity gradient of the sky (el Jundi et al. 2014), and, as an apparent ‘final resort’, the chromatic gradient (el Jundi et al. 2015a). The compass system of nocturnal beetles follows another order, with the polarised light pattern (rather than the moon) as the primary cue (Dacke et al. 2003a; el Jundi et al. 2015b; Smolka et al. 2016). Interestingly, if coaxed into rolling their balls during the day, nocturnal beetles switch to the hierarchy of a day active beetle and orient instead to the sun as their primary cue of reference (el Jundi et al. 2015b). This suggests that the hierarchy of compass cues within the backup system of the South African beetles is dynamic, and allied to the visual ecology of the navigator.

Studies of celestial orientation in dung beetles have so far focused on South African ball rolling, savannah-living beetles that primarily orient under open, blue skies. However, ball-rolling beetles are found on all continents of the globe (except for the Antarctic), in habitats ranging from deserts to rainforests (Cambefort 1991; Scholtz et al. 2009). The visual environment of woodlands and forests differs from that of a savannah in many ways, with a core difference being the extent of overhead vegetation (Endler 1993; Shashar and Cronin 1998). The denser the canopy, the more frequently the sun will be hidden from the direct view of ground-dwelling animals. Provided that patches of clear sky are discernible, a wide-field cue, such as polarised skylight, will, however, remain equally reliable under a canopy, as under the open sky (Shashar and Cronin 1998; Hegedüs et al. 2007). Here, we examine the straight-line orientation strategy of the woodland-living dung beetle, *Sisyphus fasciculatus*, to consider how the compass system of this species is influenced by its visual ecology.

## Methods

### General

With the aid of dung-baited pit-fall traps [plastic container (1 l) covered by a metal grid (30 × 30 cm)] (Tocco et al. 2017), two diurnal South African dung beetle species, *Si. fasciculatus* and *Scarabaeus* (*Kheper*) *lamarcki*, were collected on Pullen nature reserve (31.10°E, 25.34°S) and Stonehenge game farm (24.32°E, 26.39°S), respectively.

To determine the vegetation type of the savannah woodland in which *Si. fasciculatus* is most abundant, three pit-fall traps were placed in the predominantly open region (dominant grass species; *Heteropogon contortus, Sporobolus pyramidalis* and *Chloris pycnothrix*) and three were placed in the predominantly closed region (dominant tree species; *Sclerocarya birrea, Searsia pentheri* and *Erythrina lysistemon*) of the beetles’ habitat for three non-consecutive sample occasions during March 2018. The traps were placed along a transact 50 m apart, and baited at ground level using 400 g of fresh cow dung per trap. Beetles falling into the traps were killed by a 30% aqueous solution of ethylene glycol. Traps were emptied and re-baited with fresh dung every 6 h during daylight.

All behavioural data recorded from the field were collected in the same locations as given above, under clear skies, at solar elevations ranging between 45° and 60°, during November 2017 and March 2018. Experiments were recorded using a Sony Handycam HDR-CX730E (fitted with a 0.42 × wide-angle lens) mounted from above with the lens facing downwards. Circular statistics on measured data was performed using Oriana 3.0 (Kovach Computing Services, Anglesey, UK). All circular data are reported as mean ± circular standard deviation. All linear data are reported as mean ± standard error of the mean. The angular distribution of the change in bearings was tested using the *V* test with the expected mean of 0°, with the exception of the ersatz sun test experiment where the expected mean was determined to 180°. If the distribution of change in bearings was directed around the expected mean, the *V* test was significant.

### Determining step size

Under a full view of the sky, on a flat, sand-coated, wooden surface, 20 individuals per species were allowed to roll their dung ball beside a millimetre scale. From the footage of the overhead video camera, the *x-* and *y*-coordinates of the start and end points of a stride were extracted (ImageJ1©, National Institutes of Health, Bethesda, MD, USA). The length of a stride was determined as the distance from where the limb, controlling forward movement during ball rolling (hind leg for *Si. fasciculatus*, foreleg for *Scarabaeus lamarcki*), was steady on the arena surface, to when the same limb was seen to be steady on the surface again. True distances were obtained from the millimetre scale present in the frame. Five strides per beetle were measured to obtain an average step size for each species.

### Determining orientation precision

Since the precision of orientation can be expected to weaken with an increasing number of steps (Benhamou and Bovet 1992; Cheung et al. 2007), orientation precision in the two species was measured over a radial distance corresponding to a set amount of steps (20) for the respective species (*Si. fasciculatus*: 30 cm; *S. lamarcki*: 52 cm). Under an open sky, a beetle and its ball were placed in the centre of a sand-coated, circular, wooden, arena. From here, the beetle was allowed to roll to the perimeter of the arena where its exit bearing was recorded and the beetle, with its ball, was placed back in the centre of the arena again. This was repeated 20 times for each individual and recorded from above. The mean resultant vector length (*R*) of these 20 exit bearings was calculated for each individual and used as a measure of orientation precision.

The paths the beetles travelled were analysed with custom-made tracking software (kindly provided by Dr. Jochen Smolka, Lund University) in Matlab R2016a (Mathworks Inc., Natwick, MA, USA). A camera calibration software in Matlab was used to correct for optical distortions, and true distances travelled were obtained from a calibration pattern (3.9 × 3.9 cm, black and white squares) temporarily placed on the surface of each arena during data collection.

### The hierarchy of cues in the celestial compass system

A beetle was placed alongside its dung ball on a sand-coated, circular, wooden arena, with a radius of 50 cm (*solar orientation* and *simulated solar orientation*) or 30 cm (*polarisation orientation*). The beetle was allowed to roll its ball to the perimeter of the arena, where the exit bearing was noted. This marked the end of the first trial. For the second trial, the compass cue in question (see below) was changed by 180° (*solar orientation* and *simulated solar orientation*) or 90° (*polarisation orientation*) before the beetle was placed back in the centre of the arena and allowed to roll its ball to the perimeter a second time. Here, a second exit bearing was noted. Next, a third trial, presenting the same condition as the initial trial, was performed as a control for orientation performance. To determine directional changes in the test and control conditions, the difference in exit bearings between roll one and roll two (*test*) and roll one and three (*control*) was calculated. To avoid any influence of weather conditions, the two species were tested at the same time, alternately. A Mardia–Watson–Wheeler test was used to test for differences in the directional changes recorded for the two species. All directional statistics were obtained from Oriana 3.0 (Kovach Computing Services, Anglesey, UK).

#### Solar orientation

In the first trial, the beetle was placed in the centre of the arena with a full view of the sky and allowed to roll its ball to the perimeter where the bearing was recorded. For the second trial, the sun was covered from the beetle’s field of view using a wooden board (100 × 75 cm) while simultaneously changing the apparent position of the sun by 180° with the aid of a mirror (30 × 30 cm).

#### Polarisation orientation

The beetle was placed under a circular, UV-transparent polarisation filter (BVO UV Polarizer, Bolder Vision Optik©, Boulder, CO, USA) of 30 cm radius positioned in the centre of the arena, under a full view of the sky. The filter was mounted on four legs (10 cm in height), and the edge of the filter was fitted with black cloth to prevent light entering from under the filter. The initial orientation of the filter was alternated for each beetle, with half of the beetles starting the initial trial with the polarisation filter aligned with the natural polarisation band of the sky (0*°*), and the other half with the filter aligned perpendicular to the natural polarisation band of the sky (90*°*). For the second trial, the polariser was turned by 90°.

#### Simulated solar orientation

In this set of experiments, the beetle was placed in the centre of a flat, wooden, circular arena, and presented with a green unpolarised light spot (LED with emission peak around 530 nm; LZ1-00G100, LedEngin, Inc., CA, USA) at an elevation of 45°, in an otherwise completely darkened room. For the second trial, the azimuthal position of the green light spot was changed by 180°.

## Results

### General description of *Si. fasciculatus*

**Fig. 1:**
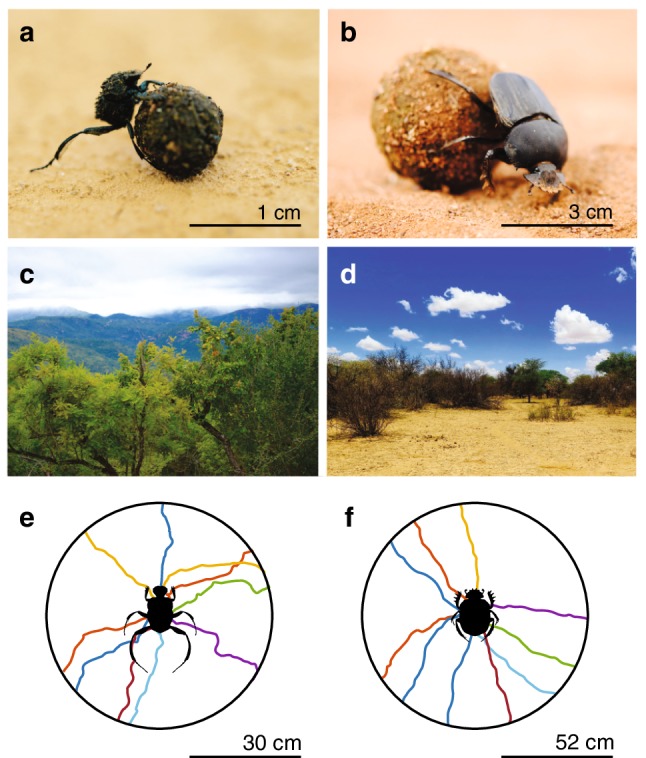
Habitat and straight-line orientation in two different dung beetle species. **a***Si. fasciculatus* and **b***S. lamarcki*, found in savannah woodland (**c**) and savannah habitat (**d**), respectively, roll their dung balls away from the dung pat on straight paths along a variety of bearings. Trajectories of ten randomly selected individuals rolling over a radial distance equivalent to 20 steps are shown for each species (**e***Si. fasciculatus*; **f***S. lamarcki*)

*Sisyphus fasciculatus* (Fig. 1a) has an average body length (tip of abdomen to tip of pronotum) of 0.5 cm ± 0.01 cm, with a pronotum width of 0.3 cm ± 0.01 cm (mean ± SEM, *N* = 20) and hind leg step size of 1.5 cm ± 0.1 cm (*N* = 20) (Fig. 1a). In comparison, its savannah-living relative *S. lamarcki* has a body length of 2.86 cm ± 0.04 cm with a fore leg step size of 2.89 cm ± 0.08 cm (Fig. 1b). A notable difference between these species is that *Si. fasciculatus* drags its ball backwards using its hind legs, whereas *S. lamarcki* pushes its ball backwards using its forelegs (Fig. 1a, b).

1175 individuals of *Si. fasciculatus* were collected within two regions of savannah woodland (Fig. 1c): open canopy area (dominated by grass) and closed canopy area (dominated by trees). In total, 821 individuals (70%) were found in the closed region, demonstrating that *Si. fasciculatus* frequently forages for dung within the closed environment of its woodland habitat.

### Orientation precision of the compass system

#### Orientation precision in *Si. fasciculatus*

The angular direction of the first bearing of each individual when rolling 20 times across the centre of a circular arena (30 cm radius) was measured and found to be randomly distributed within the population (*P*_*Si.fasciculatus*_ = 0.77, Rayleigh uniformity test, *N* = 20) (Fig. 1e).

**Fig. 2:**
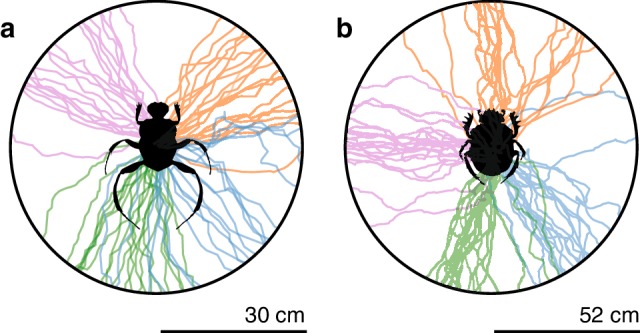
Orientation performance of two different dung beetle species. Trajectories of four randomly chosen individuals rolling 20 consecutive times over a radial distance equivalent to 20 steps under a natural sky (30 cm for *Si. fasciculatus* and 52 cm for *S. lamarcki*) are shown for **a***Si. fasciculatus* and **b***S. lamarcki*. Each colour represents the trajectories of one individual from each species

Next, the ability of *Si. fasciculatus* to repeatedly orient along its chosen bearing (Fig. 2a) was investigated by calculating the mean vector length (*R*) obtained for each individual when rolling 20 times across the centre of a circular arena (30 cm radius), resulting in an overall mean vector length of 0.90 ± 0.02 (*N* = 20) for the population (Fig. 2a).

#### Orientation precision in *S. lamarcki*

Similar to above, the exit bearings for *S. lamarcki* were randomly distributed in all directions (*P*_*S.lamarcki*_ = 0.45, Rayleigh uniformity test, *N* = 20) (Fig. 1f). *S. lamarcki* were equally as capable of maintaining a constant bearing direction over consecutive rolls as *Si. fasciculatus,* with a mean vector length for the population of *R* = 0.91 ± 0.02 (*N* = 20) (Wilcoxon rank sum test; *P* = 0.30, *N* = 20) (Fig. 2b).

### The hierarchy of cues in the celestial compass system

#### The role of the sun in the celestial compass system of *Si. fasciculatus*

**Fig. 3:**
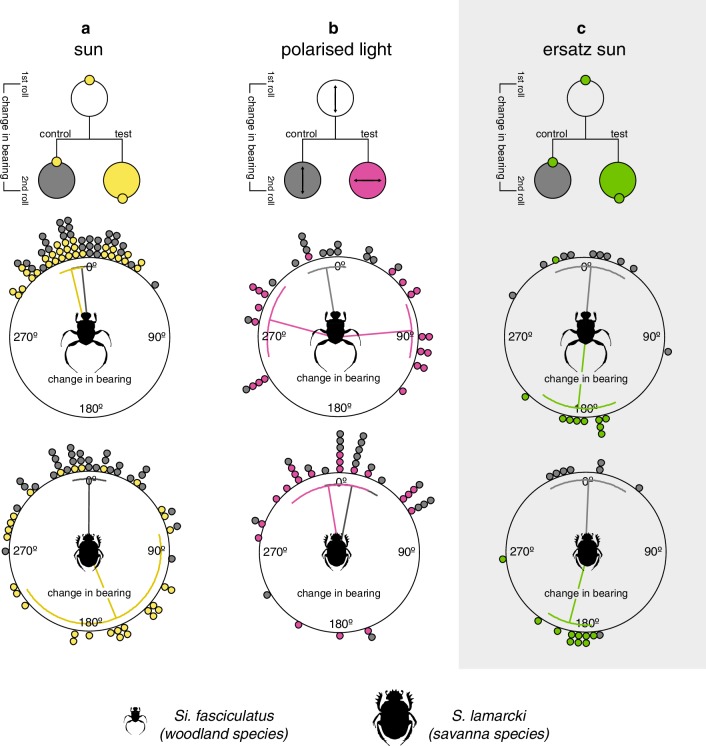
Response to directional changes of compass cues. *Si. fasciculatus* and *S. lamarcki* were allowed to roll their dung balls to the perimeter of a circular arena under an open sky (**a, b**) or in the presence of an artificial sun in an indoor lab (**c**). When the beetle reached the periphery of the arena, the exit angle was noted and the beetle was placed back at the centre again, now with the test cue (sun, polarised light or artificial sun) moved to a different position. **a** The apparent position of the sun was changed by 180° using a mirror; **b** the apparent e-vector direction of the celestial polarisation pattern was turned 90° with a polarising filter; **c** the position of the ersatz sun (green light) was switched by 180°. The difference between the two exit angles defines the response to the treatment (**a** 180° change in sun position, yellow circles; **b** 90° change in the direction of polarisation, magenta circles; **c** 180° change in ersatz sun position, green circles). Under the mirrored sun, *S. lamarcki* responded by a change in exit bearing approaching 180° (yellow line), while *Si. fasciculatus* showed no significant change in bearing (yellow line). When turning the e-vector by 90°, *Si. fasciculatus* showed a clear response (magenta line), while *S. lamarcki* did not respond to this treatment (magenta line). Both species responded with a change approaching 180° (green line) in response to a 180° change in position of the ersatz sun indoors. After the second exit bearing was noted, the beetle was placed back at the centre again and allowed to exit the arena a third time, now with the cue rotated back to its initial position. The angular changes between the first and third trials (*control*) are indicated by *grey circles* in all treatments. No significant change in exit bearing was observed, which indicates that beetles attempted to adhere to the same initial bearing throughout the experiment. Error bars represent one circular standard deviation

Next, we investigated the role of the sun in the compass system of *Si. fasciculatus*. When allowed to roll once across the arena under an unobscured sky, followed by a second time under a manipulated sky, where the sun had been mirrored by 180° and the real position of the sun was hidden from the beetle’s view (*test*), no significant change in bearing was observed (*µ*_*Si.fasciculatus*_ = 354.1° ± 24.1°, *V* test (with an expected mean of 0°); *P*_*Si.fasciculatus*_ < 0.001, *V* = 0.98, *N* = 30) (Fig. 3a, top graph, yellow data points). This suggests that *Si. fasciculatus* either does not use the sun as a compass cue, or does not use the sun as its *primary* cue for orientation. The change of bearing direction was also calculated for each individual beetle between the first roll and third roll, both made under an unobscured sky (*control*). Under this condition, the average change of bearing was around 0° (*µ*_*Si.fasciculatus*_ = 346.2° ± 27.5° (mean ± circular SD), *V* test (with an expected mean of 0°); *P*_*Si.fasciculatus*_ < 0.001, *V* = 0.87, *N* = 30) (Fig. 3a, top graph, grey data points).

#### The role of the sun in the celestial compass system of *S. lamarcki*

When *S. lamarcki* was allowed to roll once under an unobscured sky, followed by a roll under the manipulated sky (in an identical setup as for *Si. fasciculatus* above), these beetles showed a marked response to the apparent 180° change in solar azimuth, with an average change in bearing of 157.5° ± 106.5°, *N* = 30 (Fig. 3a, bottom graph, yellow data points). This change in bearing is significantly different from the lack of response recorded for *Si. fasciculatus* (Mardia–Watson–Wheeler test; *P* < 0.001, *N* = 30, *W* = 29.8) (Fig. 3a). Similar to *Si. fasciculatus, S. lamarcki*, displayed no change in bearing direction when rolling repeatedly under an unobscured sky (*µ*_*S.lamarcki*_ = 0.5° ± 38.294°, *V* test (with the expected mean of 0°); *P*_*S.lamarcki*_ < 0.001, *V* = 0.87, *N* = 30) (Fig. 3a, bottom graph, grey data points).

#### The role of dorsal polarised light in the celestial compass system of *Si. fasciculatus*

In the next set of experiments, each beetle rolled twice under a polarising filter in the presence of the sun, with the filter rotated by 90° between rolls. Under these conditions, *Si. fasciculatus* changed their bearing by 82.8° ± 30.5° (*N* = 20), suggesting this species orientates to a dorsal pattern of polarised light (Fig. 3b, top graph, magenta data points). When instead rolling two times consecutively under the polarising filter when held in place (*control*), no significant change in direction was observed (*µ*_*Si.fasciculatus*_ = 350.7° ± 35.5°, *V* test (with an expected mean of 0°); *P*_*Si.fasciculatus*_ < 0.001, *V* = 0.07, *N* = 20) (Fig. 3b, top graph, grey data points).

#### The role of dorsal polarised light in the celestial compass system of *S. lamarcki*

When the same test was repeated with *S. lamarcki*, these beetles only changed their direction by 45.3° ± 49.7° (Fig. 3b, bottom graph, magenta data points), which was significantly smaller than the change in direction recorded for *Si. fasciculatus* (Mardia–Watson–Wheeler test; *p* < 0.001, *W* = 14.421, *N* = 20). Again, there was no significant change in the bearing direction between two consecutive rolls when the polarisation filter remained in the same orientation (*control*) (*µ*_*S.lamarcki*_ = 10.83° ± 49.3°, *V* test (with an expected mean of 0°); *P*_*S.lamarcki*_ < 0.001, *V* = 0.68, *N* = 20) (Fig. 3b, bottom, grey line).

#### *Sisyphus fasciculatus* can orient to an ersatz sun indoors

To investigate if *Si. fasciculatus* is able to maintain its bearing direction using a single point-light source (such as the sun) as a compass cue, individuals were presented with a green light spot as an ersatz sun (el Jundi et al. 2015a) in an indoor arena in the absence of other visual cues. When maintained in the same position over two consecutive rolls (*control*), no significant change in direction between the two rolls could be observed (*µ*_*Si.fasciculatus*_ = 5.1° ± 39.9°, *V* test (with the expected mean of 0°); *P*_*Si.fasciculatus*_ < 0.001, *V* = 0.78, *N* = 10) (Fig. 3c, top graph, grey data points), indicating that *Si. fasciculatus* is able to maintain its bearing with reference to a single point-light source if this is the only cue available. When the position of the ersatz sun was changed by 180° between two rolls, *Si. fasciculatus* changed its bearing accordingly (185.9° ± 41.7°, *N* = 10, *V* test (with the expected mean of 180°); *P*_*Si.fasciculatus*_ ≤ 0.001, *V* = 0.77, *N* = 10) (Fig. 3c, top graph, green data points). In contrast to the large spread in bearings recorded for *Si. fasciculatus* outside (Fig. 1e), the spread of bearings travelled indoors was significantly clustered (*P*_*Si.fasciculatus*_ = 0.026, Rayleigh uniformity test, *N* = 20) with a mean of 24.8° ± 65.33° (mean ± circular SD) relative to the azimuth of the ersatz sun. That is, the beetles could be observed to travel along bearings in the direction of the ersatz sun.

#### *Scarabaeus lamarcki* can orient to an ersatz sun indoors

When tested in the same indoor arena as above, with the ersatz sun maintained in position, also *S. lamarcki* maintained their bearings between rolls (*µ*_*S.lamarcki*_ = 2.4° ± 43.2°, *V* test (with the expected mean of 0°); *P*_*S.lamarcki*_ < 0.001, *V* = 0.75, *N* = 10) (Fig. 3c, bottom graph, grey data points). In addition, these beetles responded to a 180° change in “solar position” by a similar change in bearing as *Si. fasciculatus* (194.9° ± 24.7°; *N* = 10) (Mardia–Watson–Wheeler test; *P* = 0.31, *W* = 2.36, *N* = 10) (Fig. 3c, bottom graph, green data points). To investigate if also the bearing directions of *S. lamarcki* were directed towards the position of the ersatz sun, the angular direction of the first bearing of each individual was measured. In this species, the bearings taken indoors were randomly distributed within the population (*P*_*S.lamarcki*_ = 0.35, Rayleigh uniformity test, *N* = 20), showing no significant difference from the distribution of roll bearings travelled under the natural sun (Mardia–Watson–Wheeler test; *P* = 0.28, *W* = 2.52, *N*_Greenlight_ = 10, *N*_Sun_ = 20).

## Discussion

### Bearing directions of* Si. fasciculatus* and* S. lamarcki*

Despite a large difference in body size and their rolling techniques [where the savannah woodland species, *Si. fasciculatus, drags* its ball backwards using its hind legs for traction (Fig. 1a), and the savannah species, *S. lamarcki, pushes* its ball backwards with its forelegs in contact with the ground (Fig. 1b)], both species move away from the centre of the arena (i.e., the dung pat) in straight lines with similar orientation precision (Figs. 1e, f, 2). The initial bearings travelled by different individuals were randomly distributed in all the directions for each species, clearly demonstrating that neither *Si. fasciculatus*, nor *S. lamarcki*, use a certain species-specific direction when orienting away from the pat, but select their bearing direction on an individual level. For *S. lamarcki*, this bearing is reset when a new ball is made, after which the beetle can be observed to roll along a different bearing (Baird et al. 2010). Whether this is also the case for *Si. fasciculatus* remains to be investigated.

#### Different strategies for compass cue integration

When the band of polarised light was set in conflict to the position of the sun, *Si. fasciculatus* turned in accordance to the 90° rotation of the polariser, while *S. lamarcki* showed a significantly weaker response to this manipulation. When the sun was mirrored, *Si. fasciculatus* did not respond to the positional change of this compass cue (Fig. 3a, top). This stands in contrast to past studies on the compass system of other diurnal, savannah-living, ball-rolling dung beetles that all (including the present observation of the solar compass in *S. lamarcki*) show a large change in rolling bearing in response to a manipulation of the sun’s position (Byrne et al. 2003; Dacke et al. 2014; el Jundi et al. 2015b).

The savannah woodland biome, inhabited by *Si. fasciculatus* (Paschalidis 1974), differs greatly from that of the open savannah, inhabited by *S. lamarcki* (Ospina-Garcés et al. 2018), with a greater tree density and a more closed canopy in the woodland (Fig. 1c, d). In addition, *Si. fasciculatus* frequently forages within the closed region of its environment (dominant tree species; *S. birrea, S. pentheri*, and *E. lysistemon*, see “Methods”). The higher annual rainfall in this biome compared to the savannah (Paschalidis 1974; Rutherford et al. 2006) also suggests a higher occurrence of clouds. While overhead vegetation and clouds will hinder the use of a solar compass, the celestial polarisation pattern will remain visible under the forest canopy (Shashar and Cronin 1998; Hegedüs et al. 2007) as well as underneath clouds, if portions of the sky can be glimpsed (Pomozi et al. 2001). Consequently, the celestial polarised light pattern is likely to be the more reliable compass cue in this type of environment.

Under the appropriate circumstances, the compass system of the woodland-living beetle is also able to obtain directional information from a point-light source. When presented with a single green light spot, a valid replacement for the real sun to a beetle (el Jundi et al. 2015a), *Si. fasciculatus* and* S. lamarcki* changed their bearings according to the azimuthal displacement of this light (Fig. 3c). The bearings chosen by *Si. fasciculatus* in response to the ersatz sun were, however, primarily directed *towards* the green light, while *S. lamarcki* could be observed to exit the arena along randomly distributed bearings (but see el Jundi et al. 2015a, 2016). This suggests that, under these laboratory conditions, *Si. fasciculatus* adapted a positive phototaxis rather than the menotactic behaviour observed outdoors (Fig. 1e). While more detailed investigations are required to determine if *Si. fasciculatus* can use the sun as a compass cue when orienting outdoors, we can conclude that the primary celestial cue for orientation differs between *Si. fasciculatus* and *S. lamarcki*.

In summary, this and the previous studies of the compass system in dung beetles suggest that the hierarchy of celestial cues varies with the visual ecology of the species. This appears to be true for species from different biomes (Buhlmann et al. 2011), as well as for a single species when night turns into day (el Jundi et al. 2015b). If the primary cue within each system is also the cue that supplies the compass with the highest degree of precision, it will be the focus of our next study.

#### Guided movement in cluttered environments

While a bare environment, such as a salt pan or a desert, can be nearly void of landmarks, the amount of tall vegetation in forests and savannah woodlands provides a large range of terrestrial cues that can be used for directional information (Hölldobler 1980; Hironaka et al. 2008; Reid et al. 2011; Rodrigues and Oliveira 2014). Not surprisingly, forest-living ants and bees rely heavily on terrestrial cues when finding their way back home (Warrant et al. 2004; Fleischmann et al. 2018a, b). In parallel, the sub-social shield bug and the African stink ant will change their bearings in response to a rotation of an artificial canopy pattern (Hölldobler 1980; Hironaka et al. 2008). Even though not directly manipulated in this study, we do not see any indication that the savannah woodland beetles stabilise their course in relation to the rich visual scenery around them. This supports earlier studies on the compass system of the dung beetles which have been shown to ignore landmarks for straight-line orientation (Dacke et al. 2013b). An important distinction between ball-rolling dung beetles and the homing insects considered above is that, instead of repeatedly finding their way back to a well-known point in space in the form of a nest, the beetles rather set and follow a once-off course towards an unknown goal in an unfamiliar terrain to bury their ball. For such a task, landmarks have little value and provide no guidance. Instead, the compass of the woodland-living beetle *Si. fasciculatus* relies on polarised skylight as its dominant directional cue when negotiating its vegetated surroundings. Further studies of the compass system of woodland living beetles will investigate if this strategy is widely adopted by straight-line orienting insects foraging in cluttered environments.
